# The projected exposure and response of a natural barrier island system to climate-driven coastal hazards

**DOI:** 10.1038/s41598-024-76749-4

**Published:** 2024-10-28

**Authors:** Jennifer A. Thomas, Patrick L. Barnard, Sean Vitousek, Li H. Erikson, Kai Parker, Kees Nederhoff, Kevin M. Befus, Manoochehr Shirzaei

**Affiliations:** 1 Pacific Coastal and Marine Science Center, U.S. Geological Survey, Santa Cruz, CA USA; 2 Deltares USA, Silver Spring, Maryland, MD USA; 3 Department of Geosciences, University of Arkansas, Fayetteville, AR USA; 4 Department of Geosciences, Virginia Tech, Blacksburg, VA USA; 5 Institute for Water, Environment and Health, United Nations University, Hamilton, ON Canada

**Keywords:** Climate-change impacts, Natural hazards

## Abstract

Accelerating sea level rise (SLR) and changing storm patterns will increasingly expose barrier islands to coastal hazards, including flooding, erosion, and rising groundwater tables. We assess the exposure of Cape Lookout National Seashore, a barrier island system in North Carolina (USA), to projected SLR and storm hazards over the twenty-first century. We estimate that with 0.5 m of SLR, 47% of current subaerial barrier island area would be flooded daily, and the 1-year return period storm would flood 74%. For 20-year return period storms, over 85% is projected to be flooded for any SLR. The modelled groundwater table is already shallow (< 2 m deep), and while projected to shoal to the land surface with SLR, marine flooding is projected to overtake areas with emergent groundwater. Projected shoreline retreat reaches an average of 178 m with 1 m of SLR and no interventions, which is over 60% of the current island width at narrower locations. Compounding these hazards is subsidence, with one-third of the study area currently lowering at > 2 mm/yr. Our results demonstrate the difficulty of managing natural barrier systems such as those managed by federal park systems tasked with maintaining natural ecosystems and protecting cultural resources.

## Introduction

**Fig. 1:**
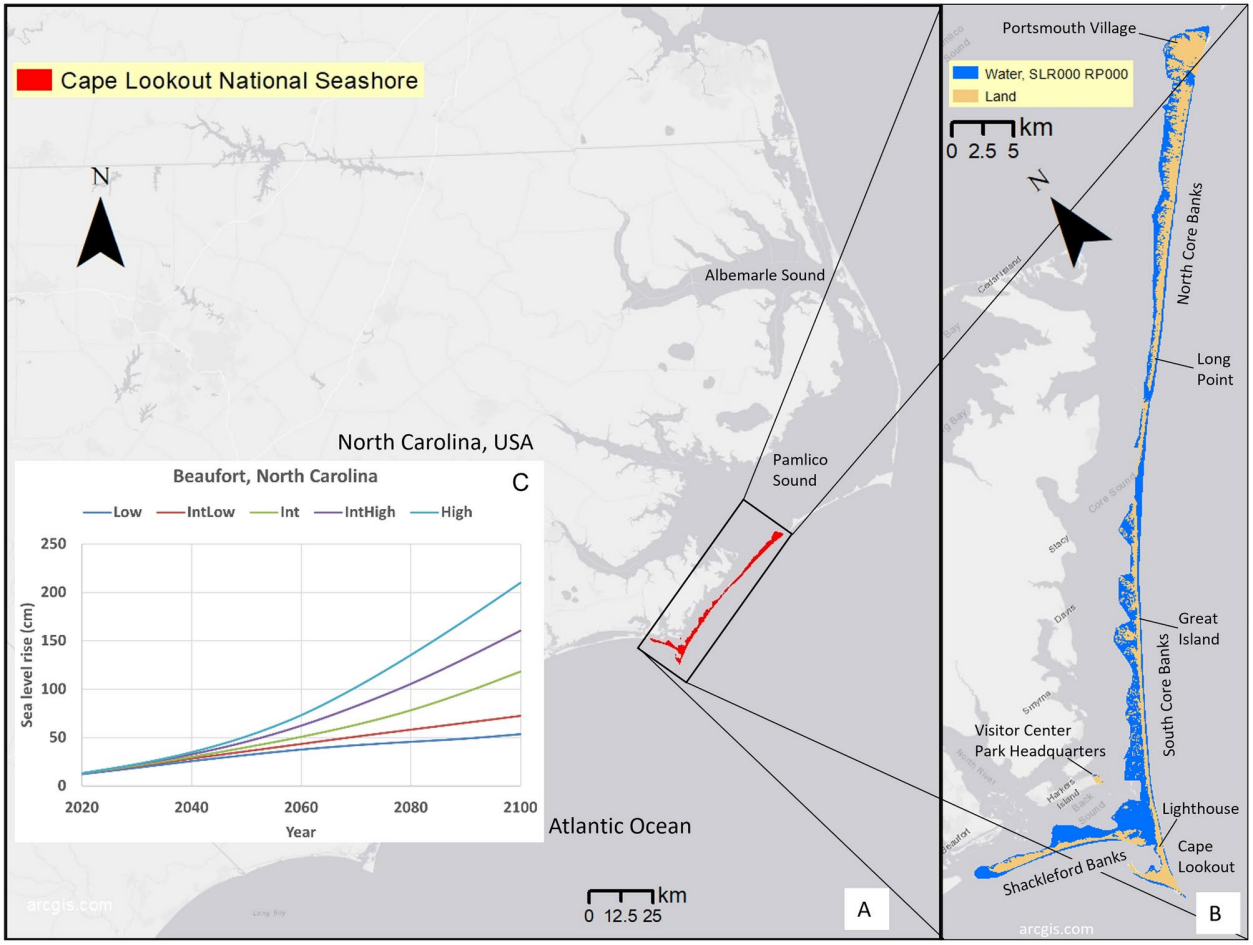
Map of study area. Panel (**A**) shows the current legislated area of Cape Lookout National Seashore (CLNS) in red. This boundary defines the extent of all data presented in this paper. Panel (**B**) shows rotated inset of boxed area shown in panel (**A**), with modeled flood results for the 0 m SLR, no storm case showing what we define as present day land (tan areas) and water (blue areas). Panel (**C**) shows sea level rise estimates from the U.S. Sea Level Rise and Coastal Flood Hazard Scenarios and Tools Interagency Task Force (Sweet et al., 2022), which projects higher than global rates of SLR for the study area (i.e., using the Beaufort tide gauge as a proxy) of 32–51 cm by 2050 and 54–210 cm by 2100. We created the maps using ESRI ArcMap 10.8.1 software http://desktop.arcgis.com/en/arcmap.

Sea level rise (SLR) poses a major threat to both the natural and built environment through increasing rates of coastal flooding, erosion, and shallow groundwater rise^1–3^. When storms occur in the future with elevated sea level, dynamic processes such as waves and storm surge will reach higher land elevations along the coast. Barrier islands, such as those that make up our study area, Cape Lookout National Seashore (CLNS) in North Carolina, U.S. (Fig. 1), buffer storm impacts for the mainland coast. However, the barrier islands themselves are at an increased risk compared to mainland coasts, as they can experience the hazards of SLR and storms on all sides. Vulnerable barrier islands can lead to more vulnerable mainland coastlines in the future^4^.

Global sea level has risen by 3.4 mm/yr during the satellite era (1993–2022)^5^ and the rate has increased by 29% to 4.4 mm/yr over the last decade (2013–2022)^6^, more than triple the 20^th^ Century rate^7^. The U.S. East Coast north of Cape Hatteras, North Carolina (just north of the study area) has been a hotspot for SLR, with a rate of increase 3–4 times higher than the global average from 1950–2009^8^. South of Cape Hatteras, sea level also rose greater than 20 mm/yr from 2011–2015, linked to large-scale ocean dynamics such as the variability of the North Atlantic Oscillation^9,10^ and/or Florida current dynamics^11,12^. At the nearest tide gauge to CLNS, in Beaufort, North Carolina, the sea level has risen by 3.4 mm/yr from 1952–2022, with notable recent acceleration^13^. In line with higher observed rates along the North Carolina coast, the U.S. Sea Level Rise and Coastal Flood Hazard Scenarios and Tools Interagency Task Force projects higher than global rates of SLR for the study area (i.e., using the Beaufort, North Carolina, tide gauge as a proxy) of 32–51 cm by 2050 and 54–210 cm by 2100 (Fig. 1C). The observation-based projections in this location are currently following the intermediate-high emission scenario, which would yield a median SLR projection of 46 cm by 2050 and 160 cm by 2100^14^.

Due to higher rates of SLR in the region, annual rates of high tide flooding have been increasing rapidly over the last several decades^15^ and are expected to increase by up to an order of magnitude by 2050^14^. Recent hurricanes have had major impacts (i.e., extensive flooding, erosion, loss of habitats) on the region, including Matthew (2016), Florence (2018), and Dorian (2019). For coastal North Carolina, flood risk for extreme storm events doubles with just 5–10 cm of SLR^16^, and therefore, in the coming decades, hurricanes will likely cause more severe impacts than in the recent past.

Barrier islands can rapidly evolve in response to changing environmental conditions such as SLR and storms^17^. For example, barrier islands migrate landward as sea level rises, which can help these systems persist and thus continue protecting the mainland coasts behind them^4,18^. For developed barrier islands, natural responses are often managed to protect communities and infrastructure, but altering natural processes can be less sustainable for a barrier island in the long term^4,19–22^. On the barrier islands of CLNS—North Core Banks, South Core Banks, and Shackleford Banks (Fig. 1)—, natural processes such as island migration and overwash are typically allowed to occur^23^.

The many hazards that can arise from SLR and storms on CLNS, including flooding, groundwater rise, erosion, and island migration, make managing these islands challenging. There are numerous resources to manage on CLNS: e.g., historic villages, a historic (and still operational) lighthouse, camping facilities, ferry landings, and nesting and habitat areas for several federally protected and/or threatened species. Managers need the best possible long-term projections of potential changes from SLR and storms that affect their managed assets.

### Study approach

To provide some of these long-term projections for CLNS, here we assess the exposure of this natural barrier island system (Fig. 1) to four different types of hazards: modeled projections of flooding, groundwater rise, and shoreline change caused by SLR and storms over the 21st Century, and vertical land motion from 14 years of historical observations. CLNS consists of the largely undeveloped barrier islands North Core Banks, South Core Banks, and Shackleford Banks, and the eastern end of developed Harkers Island (which is inland of the barrier islands and is not a barrier island), where the Visitor Center is located (Fig. 1). We show that the viability of this barrier island system will be compromised by increasingly severe flooding, rising groundwater, erosion, and land subsidence over the next century. As part of the National Park Service, these islands serve as a global proxy for a common coastal management dilemma in the face of significant present-day and future coastal hazards: protecting important cultural and archaeological resources while seeking to allow natural processes to continue, and/or favoring nature-based interventions for coastal sustainability.

The data used in this paper come from results from the Coastal Storm Modeling System (CoSMoS)^24–27^ approach and satellite observations applied to the U.S. Southeast Atlantic coast, which provide future coastal hazard data for the area from Norfolk, Virginia to Miami, Florida, from the coast to typically about 100 km inland to 10 m topographic elevation^28,29^. Thus, analyses like those presented in this paper could be performed along any other region in this area where this data is available, including along developed barrier island systems. The datasets used are available from Barnard et al.^28^ and detailed in the Data Availability section.

For each of the four hazards presented, we took the data for the full Southeast region and reduced it to just the data that falls within the boundaries of CLNS (Fig. 1, colored regions). Then we quantified areas and mapped patterns of the hazards within CLNS. The model results are presented for a range of SLR scenarios intended to encompass plausible projections by 2100 (0, 0.25, 0.5, 1.0, 1.5, 2.0, and 3.0 m). The flood model projections also include a range of storm conditions across each SLR projection (no-storm, 1-year, 20-year, and 100-year return period storms). In this paper, we present results relative to SLR values, not time, to decouple the uncertainty of timing from future SLR. However, in the Discussion section, we relate results to time, using the SLR values closest to local estimates for the intermediate-high scenario for the years 2050 (0.46 m) and 2100 (1.60 m)^14^.

It is important to note that the four hazard projections are not coupled. That is, neither shoreline change nor vertical land motion results are applied to the topobathymetry or morphology for the other products; present-day topobathymetry is used for flooding and groundwater modeling. Island morphological profile changes, including changes in dune height, due to SLR or storm impacts are not modeled, thus only present-day topography and bathymetry are used. The assumption of static morphology within the flooding and groundwater models implies that their results are most pertinent in the shorter term or near the Visitor Center on developed Harkers Island (the non-barrier island) (Fig. 1), where static morphology is more likely.

However, morphology will inevitably change over time. In the longer term, as sea level rises more and storms occur on the elevated sea level, natural barrier islands tend to migrate landward, as our shoreline change results suggest, with overwash tending to build interior elevation^30^ while the overall offshore profile roughly preserves its shape. However, recent studies suggest that barrier island retreat rates do not necessarily increase monotonically with SLR, due to the role of storms and biophysical interactions^31–33^. Despite the general tendency of storms to increase interior elevations with sea level rise, storms can sometimes cause volume loss, as seen with sound-side inundation due to Hurricane Dorian (2019) that led to elevation and volume loss through extensive erosional washout channels on North Core Banks^34^. While the shoreline change results indicate the barrier islands will migrate landward in the projected wave/storm climate, we cannot speculate on the exact morphological changes that will occur on CLNS. Additionally, the land subsidence demonstrated in our vertical land motion results will decrease land elevations on which all these processes occur.

Thus, results show the best estimates using present-day morphology and clearly demonstrate the vulnerability of this barrier island system. Specific locations of flooding or change in groundwater table depths will not be exact in the longer-term future, when the barrier island locations and morphology will have evolved in ways that depend on the coupling between the islands and their backbarrier marshes, on the timing and intensity of storm flooding, overwash events, waves, and winds, on dune structure and grasses, and on relative SLR^21,22,30,34–36^, processes that were not accounted for in the models.

Individual details on the data used for each of the four hazards—overland flooding, groundwater depths, shoreline change, and vertical land motion—are provided in the Materials and Methods section and in cited references that go into greater detail. The CoSMoS modeling approach applied was initially developed for use in California, with some modifications made for the U.S. Southeast coast. For flood modeling, modifications account for tropical cyclones and compound flooding common to the region, including pluvial, fluvial, and oceanic drivers^37–39^. Shoreline change modeling follows the same approach as Vitousek et al.^78^ using local satellite-derived shoreline observations and modified to include a range of transgression slopes, as befitting the additional uncertainty of a passive coastal margin setting^40^. Here, we present only the intermediate slope. Vertical land motion is derived from synthetic aperture radar (SAR) satellites. While uncertainties are not presented in this paper, they are included with the source data and references. For example, for shoreline change, potential storm erosion uncertainty bands were made for the same storm return periods used in the flood modeling; and though vertical land motion is not included in the topobathymetry used for the models of the other three products, it was included as part of the total uncertainty in the flood modeling (neither are presented here). See Materials and Methods for more information.

## Results

### Overland flooding

Within the boundaries of CLNS (an area of approximately 171.1 km^2^, indicated in red in Fig. 1A), the overland flood model results for no SLR and no storm (present-day, non-storm conditions) define approximately 77.6 km^2^ as present-day land (tan areas in panel B) and the remaining 93.5 km^2^ as water (blue areas in Fig. 1B), indicating the extent of high tide during a spring tide. In the model, water covering the land can come from tides, storm surge, wave setup, precipitation, and/or SLR. Inundation is used here to refer to water covering the land due to the maximum extent of the sea level with high tides, for SLR with no storms. Intermittent flooding is due to storms^41^.

**Fig. 2:**
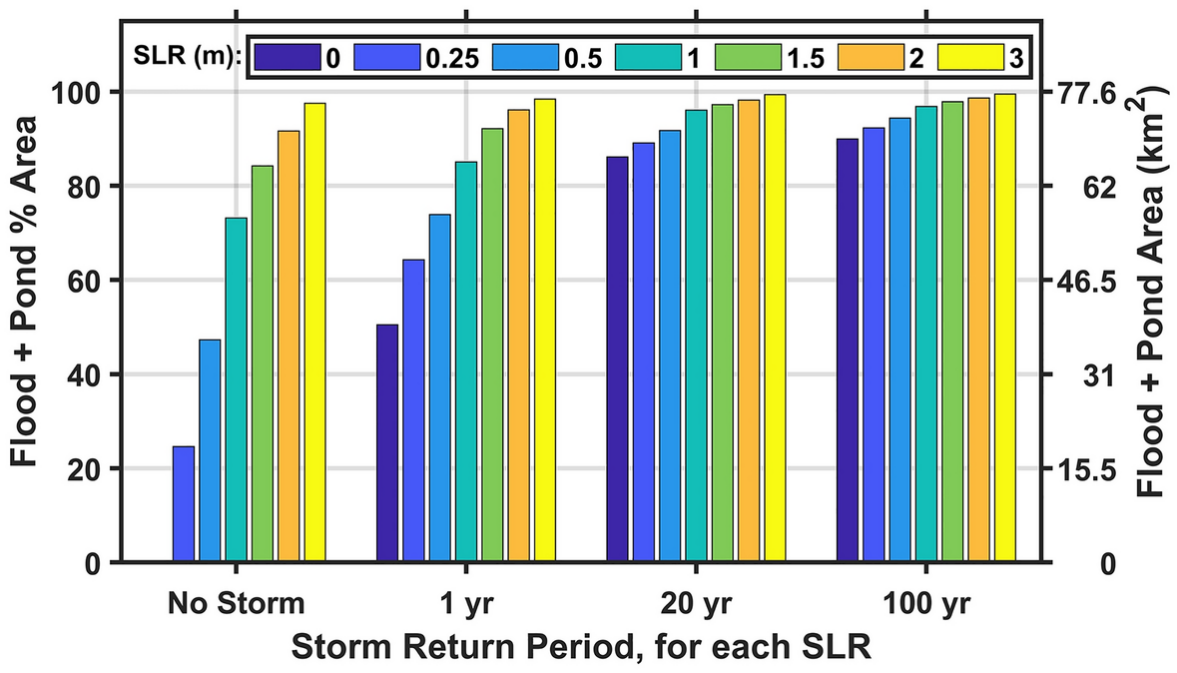
Histogram of projected flooding plus ponding over land areas of Cape Lookout National Seashore for each sea level rise (SLR, colors) and storm return period (x-axis) modeled, shown in both percent of land area (left side y-axis) and land area (km^2^, right side y-axis). No value is shown for the 0 SLR, No Storm scenario because it is used to define land versus water areas within Cape Lookout National Seashore and thus floods 0% of the land.

For the 27 other SLR and storm combinations modeled (other than the no SLR, no storm scenario), 25 result in over 50% of the present-day land area covered in water (Fig. 2). On CLNS, fall and winter low pressure systems combined with large high tides have been known to cause flooding of the ocean beach into the dunes and of the salt marsh into the backroad on the North and South Core Banks at least once per year, likely approaching 50% of the area of these islands (J. Altman, Supervisory Biologist, CLNS, personal communication, April 30, 2024). For the 20- and 100-year return period storms modeled, any SLR value, including present-day sea level (0 m SLR)—and thus, applying to present-day morphology for the 0 m SLR case—results in over 85% of the land being flooded during the storm. Hurricanes Dorian (2019) and Florence (2018) were seen to flood roughly 85% of the land, as the 20-year return period model estimates (J. Altman, Supervisory Biologist, CLNS, personal communication, April 30, 2024). Additionally, Sherwood^34^, supports this extensive flooding during Hurricane Dorian with the over 80 erosional washout channels observed stretching from the marsh to the ocean on the 36 km of North Core Banks. For the 20- and 100-year storms modeled with SLR of 1 m or more, over 96% of the present-day land is flooded.

As discussed in the Study Approach section, the flooding areas and locations may be different due to the evolution of barrier island morphology from storms and SLR. Over the course of sea level rising by 1 m, the natural barrier islands are likely to migrate landward in position, with dunes generally decreasing in height relative to sea level and overwashing more frequently, leading to maintenance of interior island elevation relative to sea level as landward migration occurs. These potential increases in interior island elevation with SLR might prevent future flooding of some island interior areas. But because the model results for the 0 m SLR case show that a 20- or 100-year return period storm occurring today would flood over 85% of the land during the storm, it is likely that with 1 m of SLR and the accompanying unknown morphological changes, the flood area during these storms could be at least as great.

Compared to the more extreme storm return periods, the no-storm and 1-year return period storm model results have a broader range of flood areas across SLRs. For example, for 0.25, 0.5, 1, and 1.5 m of SLR with no storms, 25%, 47%, 73%, and 84% of the land area would be inundated, respectively. During 1-year storm events, flooded areas increase, with a smaller spread between SLR cases than the no-storm condition, to 51%, 64%, 74%, 85%, and 92% of the land flooded for 0, 0.25, 0.5, 1, and 1.5 m of SLR, respectively. Again, with the unknown morphological changes that will increasingly occur over time as the barrier islands are allowed to naturally evolve, the flood areas and locations may be different. Thus, these results show us estimates over the present-day subaerial barrier island area and give a qualitative indication of variation between storm intensities and SLRs. Because of the relatively small range in total flood area between all modeled SLRs for 20- and 100-year storm return periods (Fig. 2), these results indicate that even with morphological changes to the islands over time, the 20- and 100-year storms will have a large flood impact. However, the larger ranges in total flood area between SLRs for the no storm and 1-year return period storm cases (Fig. 2) indicate the possibility for higher variability in actual inundated/flooded areas as the island morphology evolves.

**Fig. 3:**
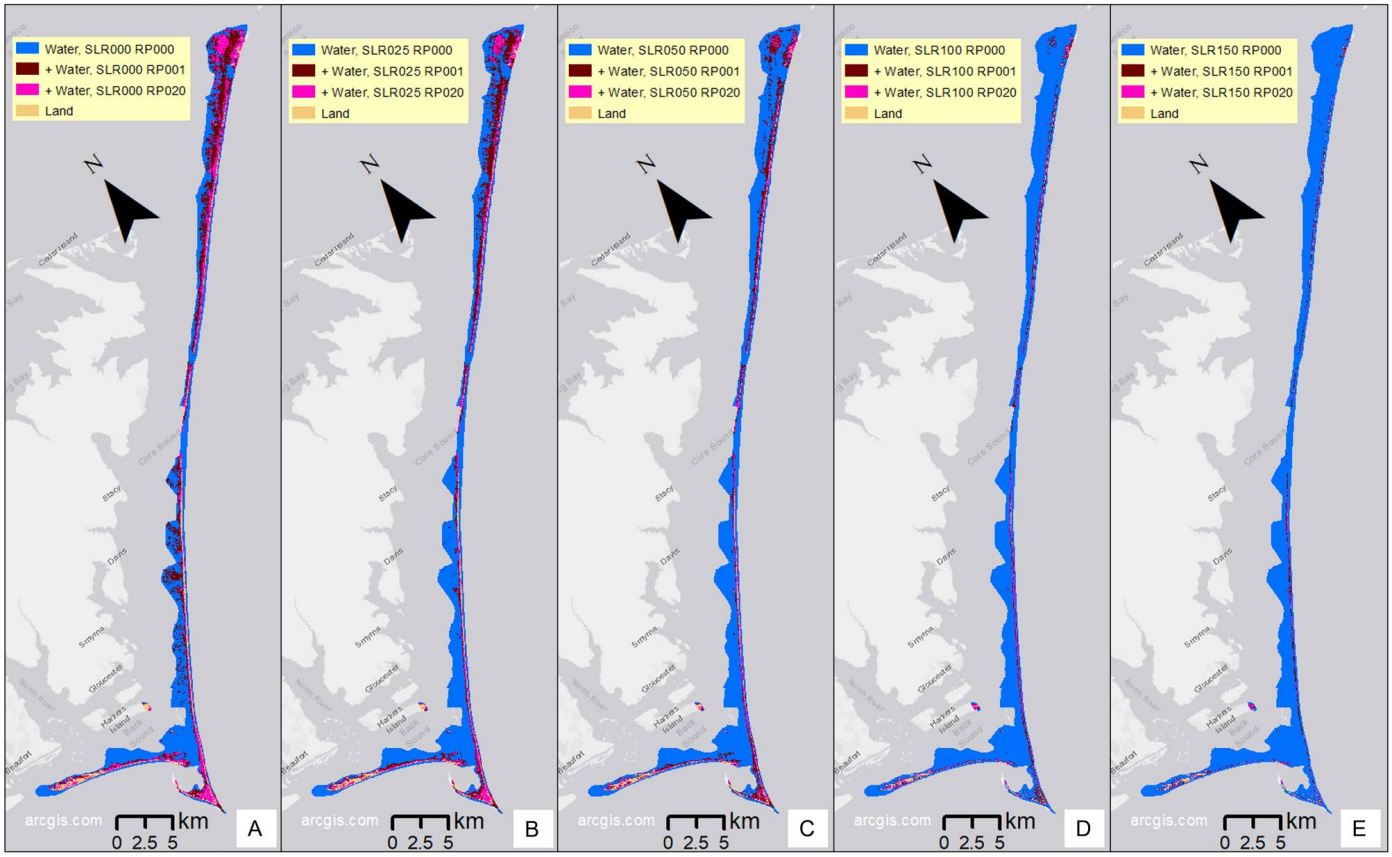
Maps of modeled flood plus pond areas over present-day island morphology, for no storm (RP000, blue), 1-year return period (RP001, maroon), and 20-year (RP020, magenta) return period storms, for no SLR (**A**); 0.25 m SLR (**B**); 0.5 m SLR (**C**); 1 m SLR (**D**); and 1.5 m SLR (**E**). Layers are additive, in that both blue and maroon together show the inundation for the 1-year return period storm; blue, maroon, and magenta together show inundation for the 20-year return period storm. We created the maps using ESRI ArcMap 10.8.1 software http://desktop.arcgis.com/en/arcmap.

Maps of the modeled inundation/flood extent on present-day island morphology for SLRs 0 through 1.5 m (Fig. 3, panels A-E), for the no-storm condition (blue) and where additional flooding would occur due to the 1-year (maroon) and 20-year return period storms (magenta), indicate that inundation/flooding of the interior parts of the islands does not occur without a storm for SLR less than 1 m (panels A-C), but with SLR of 1 m or more, many interior island areas are inundated (panels D and E, blue areas), including Portsmouth Village at the northern end of North Core Banks (Fig. 1B). Changes in morphology may alter future inundation patterns, potentially resulting in less future interior inundation than reported in Fig. 3, due to overwash increasing interior elevations relative to present-day topography. However, the overwash flow and deposition that increase the interior elevation can cause damage to developed barrier island interior areas like the historic Portsmouth Village, which experienced significant damage from flooding during Hurricane Dorian (2019)^42^. The eastern end of Harkers Island, the location of the Park Headquarters and Visitor Center (Fig. 1B), maintains a mainly dry interior for model scenarios of no-storm conditions with up to 1 m of SLR, and flooding over interior areas is limited to storm conditions. Beginning with the 1.5 m SLR model scenario, some interior areas of this part of Harkers Island are projected to experience inundation (Fig. 3E), and total inundation here occurs when SLR >  = 3 m (not shown). Because developed Harkers Island is not a barrier island, natural morphology changes like migration would not be occurring here, so it is likely that without further intervention, this area would experience these projected impacts.

### Groundwater depths

**Fig. 4:**
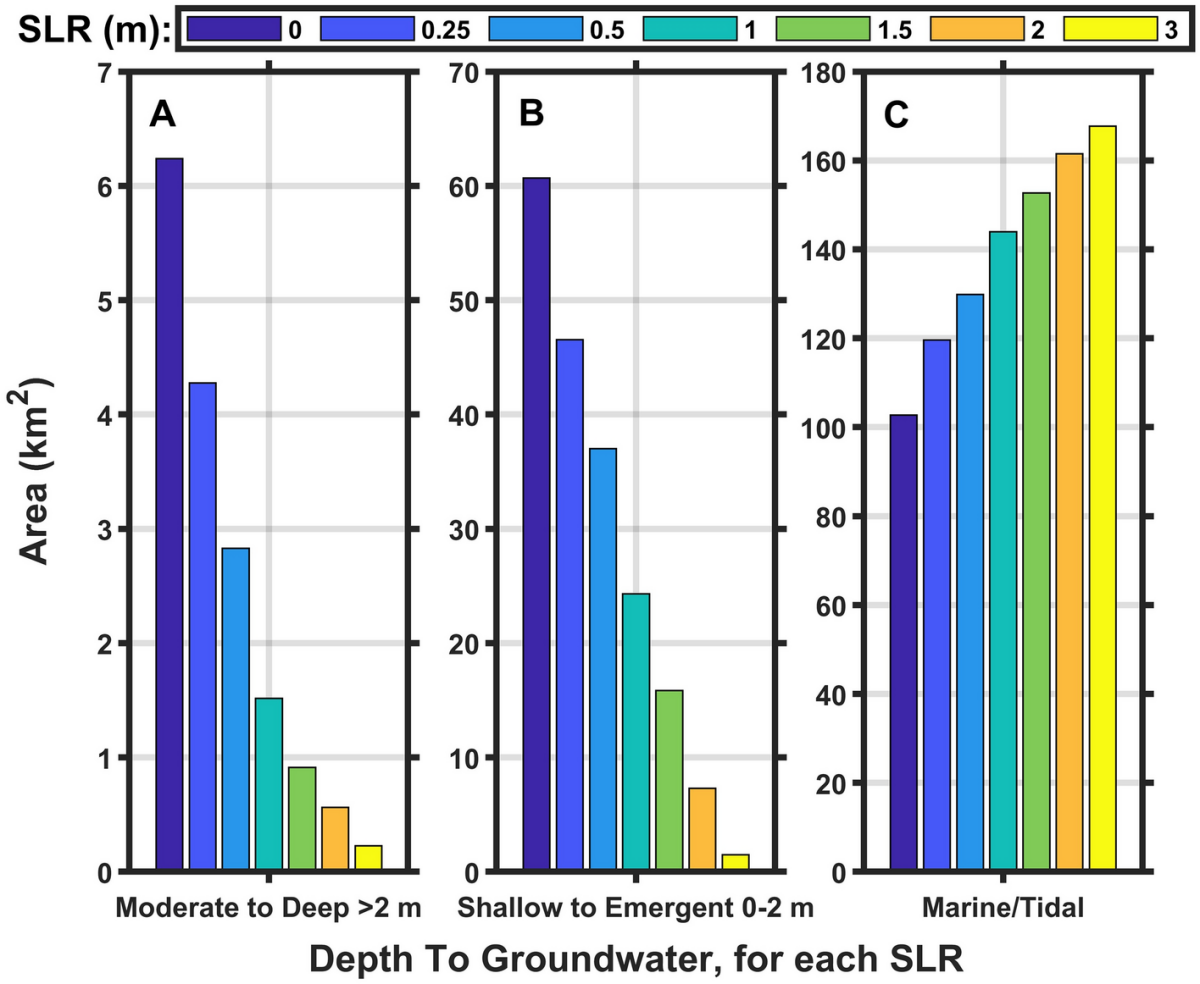
Histogram of modeled depth to groundwater showing total area of each binned depth category for Cape Lookout National Seashore for each sea level rise (SLR) scenario (no storms). Note the different y-axis scales of each panel.

**Fig. 5:**
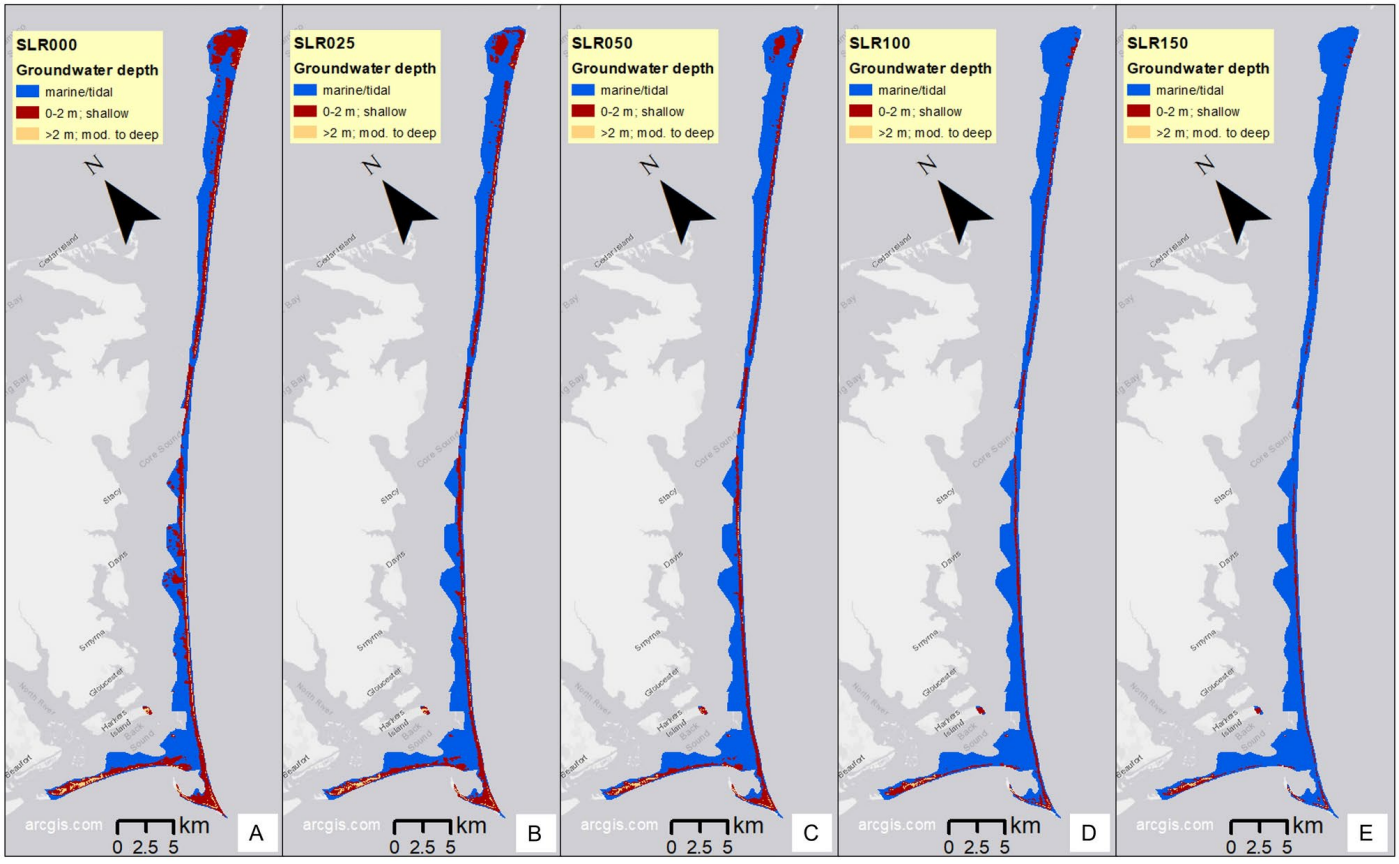
Maps of modeled groundwater depth groups for no SLR (**A**); 0.25 m SLR (**B**); 0.5 m SLR (**C**); 1 m SLR (**D**); and 1.5 m SLR (**E**) (no storms). We created the maps using ESRI ArcMap 10.8.1 software http://desktop.arcgis.com/en/arcmap.

As sea level rises, saltwater tends to intrude into the subsurface, pushing the groundwater table upward, which can lead to surface flooding with emergent groundwater and the loss of fresh groundwater, which is a vital resource to plants and animals. Model results estimate the response of the water table depth to SLR, without identifying the salinity, which may be fresh, brackish, or saline. Model results project that much of the land area of CLNS has a shallow depth to groundwater under present conditions (Figs. 4 and 5). Modeled depths of the local, spatially variable groundwater table have been binned into areas with the groundwater table deeper than 2 m (non-hazard) and shallower than 2 m (denoted as groundwater hazard due to the risks from emergent groundwater flooding the land and the loss of fresh groundwater for plants and animals)^43^, with an additional bin for surface inundation due to marine/tidal influences (Fig. 4). Groundwater table depths were modeled over an approximately 170 km^2^ area of land and water combined, almost the entire CLNS boundary (see color areas in Fig. 5).

For each SLR scenario without storm effects, the model results show more areas with a shallow depth to groundwater (0–2 m, Fig. 4B) than with a deep one (Fig. 4A), by an order of magnitude. As sea level rises, the groundwater table is pushed upward to shallower depths, decreasing the already small areas of deeper groundwater (Fig. 4A). Marine/tidal surface flooding inundates areas with seawater as quickly as SLR pushes groundwater upward, resulting in the decrease of shallow groundwater areas with increasing SLR (Fig. 4B, C and Fig. 5), rather than an increase from emerging groundwater. The National Park Service Supervisory Biologist of CLNS, J. Altman reports they do not see groundwater emerging at the surface, consistent with the model results that indicate marine inundation would occur at the same time, preventing visible emerging groundwater (personal communication, April 30, 2024). The areas and locations of future shallow groundwater may differ from those in Figs. 4 and 5, potentially with some groundwater depths maintained if interior elevations increase with SLR and storms.

### Shoreline change

**Fig. 6:**
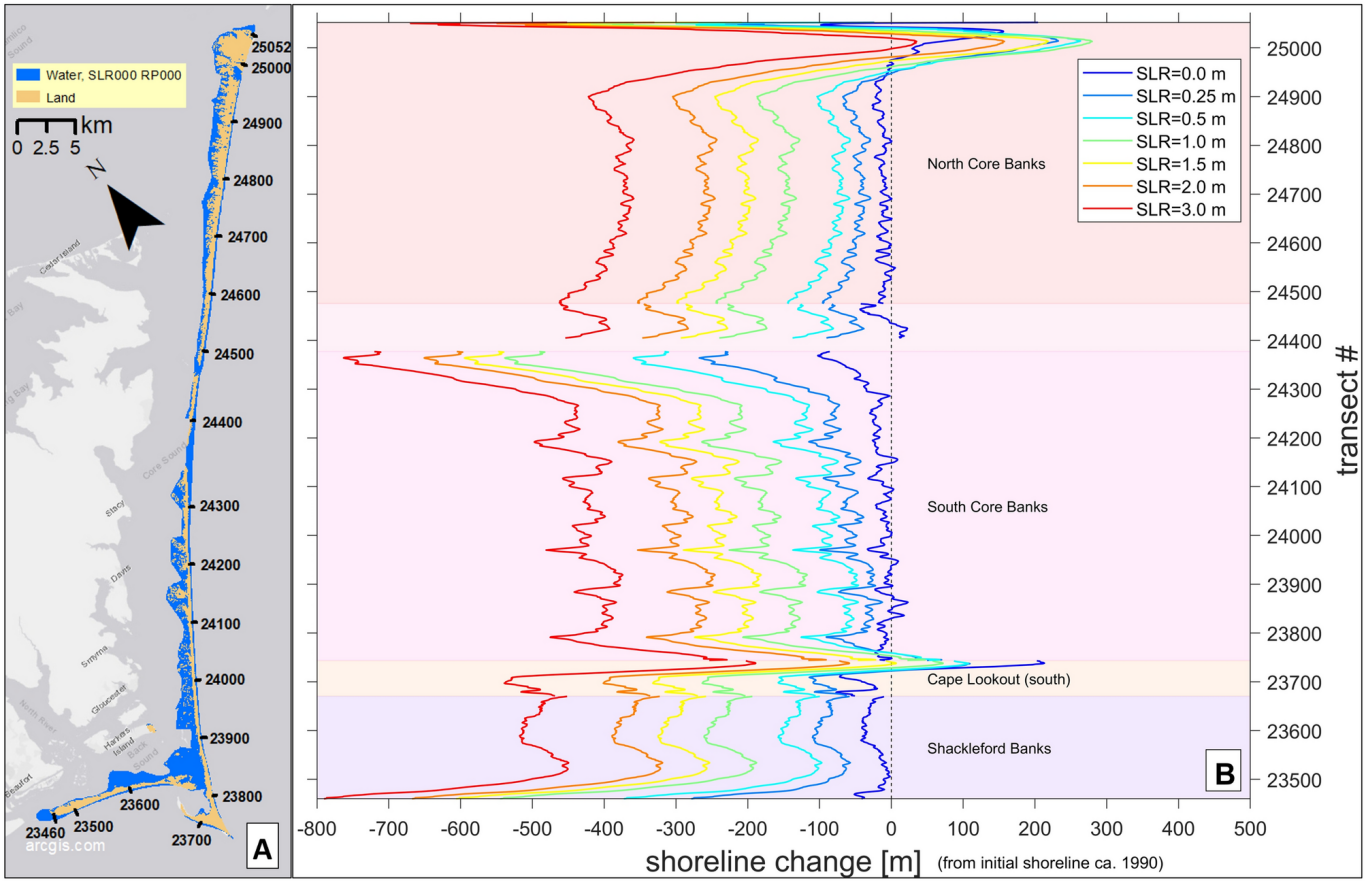
Modeled shoreline change projections for each SLR. Panel (**A**) shows present-day modeled land and water areas, with the same transect numbers indicated as in the y-axis of panel (**B**). Panel (**B**) shows shoreline change (m) relative to an initial reference shoreline location from the year 1990 (represented by the vertical dashed line along 0 m, which would demonstrate no change). (0 m SLR shows change because it is relative to that reference location from 1990.) (In the shoreline change model, SLR values were modeled in time, with the levels relating to the following years: 0 m = 2005; 0.25 m = 2038; 0.5 m = 2062; and 1 m and above = 2100.) We created the map using ESRI ArcMap 10.8.1 software http://desktop.arcgis.com/en/arcmap.

**Fig. 7:**
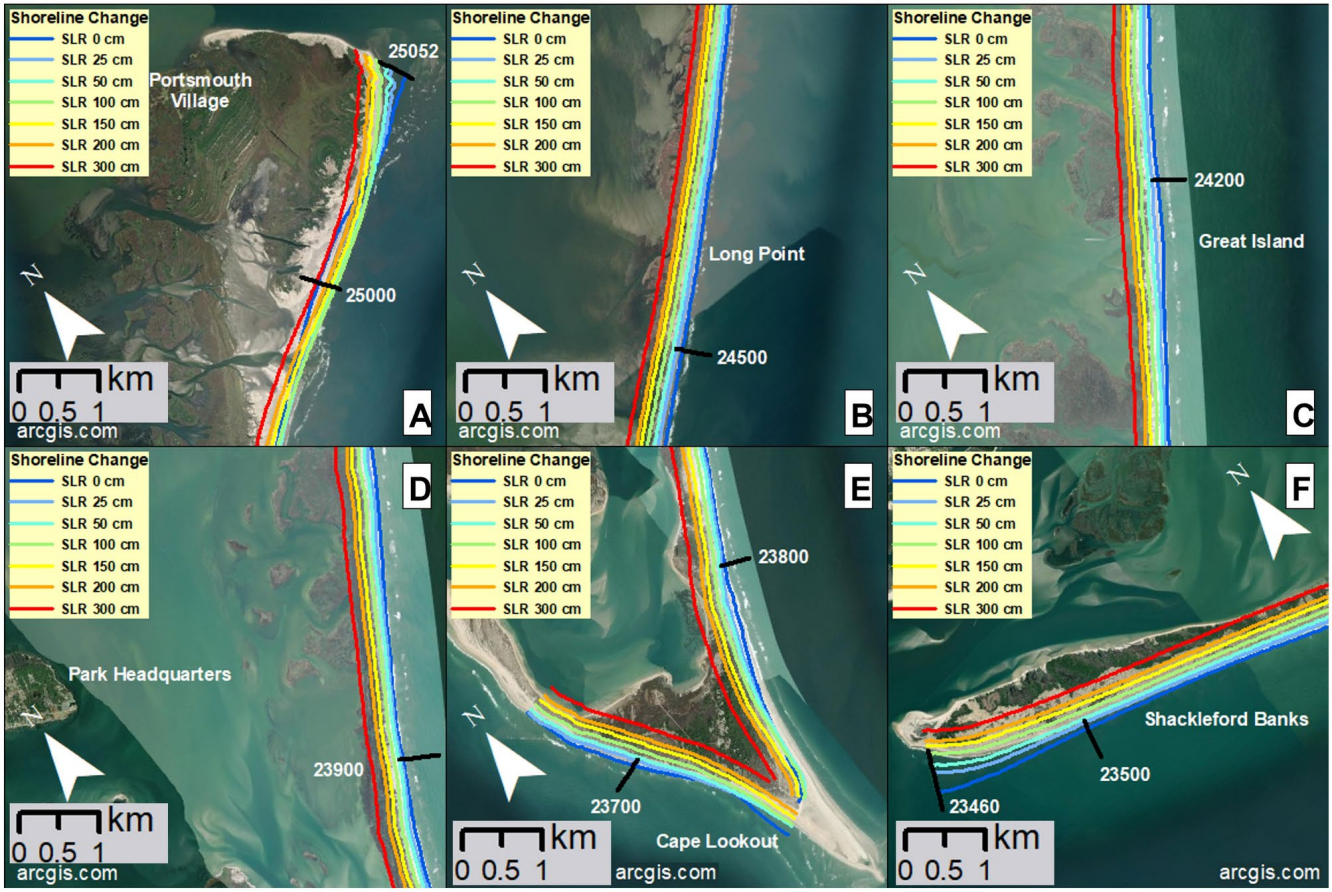
Modeled shoreline change projections overlayed onto maps of some key areas of Cape Lookout National Seashore. Note that the reference shoreline position from 1990 is not shown. Panel (**A**) shows the shoreline near the historic Portsmouth Village, at the northern extent of the islands, including a ferry terminal. Panels (**B**) and (**C**) show camp areas Long Point and Great Island, respectively, each including a ferry terminal. Panel (**D**) shows the barrier island that is oceanward of Harkers Island, the Park Headquarters location. Panel (**E**) shows Cape Lookout, which includes a ferry terminal, the Cape Lookout Lighthouse, just north of transect 23,800, and a historic U.S. Coast Guard Station. Panel (**F**) shows the western end of Shackleford Banks, including a ferry terminal. We created the maps using ESRI ArcMap 10.8.1 software http://desktop.arcgis.com/en/arcmap.

The modeled barrier shoreline of CLNS is projected to generally retreat over time with increasing SLR (Fig. 6), with average erosion values along the full region of 56 m, 92 m, 178 m, 238 m, 298 m, and 418 m for SLRs 0.25, 0.5, 1, 1.5, 2, and 3 m, respectively. However, there is significant spatial variability due to local erosion/accretion hotspots. For example, the modeled shoreline for 0 m SLR (Fig. 6, dark blue line) has accreted slightly in several locations compared to the initial reference shoreline location from the year 1990, when the model is initialized. Local accretion relative to the year 1990 occurs in a few locations in CLNS, primarily along dynamic spits and capes: e.g., for all modeled SLR scenarios at the northern extent around transect 25,000, near Portsmouth Village; and for all but 2 and 3 m SLR at Cape Lookout around transect 23,700. In these two regions, modeled shoreline retreat and accretion do not always occur in rank order of SLR (Figs. 6, 7A and 7E), due to strong local accretion trends in the area, most often due to gradients in longshore transport. For example, around transect 25,000, the modeled shoreline for 0 m SLR accretes less than all SLR cases but 3 m (driven by the persistence of accretionary trends over time), and shorelines for 0.25 and 0.5 m SLR accrete less than some higher SLRs, while just south of that accretion area, the 0 m SLR shoreline retreats more than for SLRs up to 1.5 m. Around Cape Lookout, the shoreline for 0 m SLR retreats more than for up to 1 m SLR and even retreats while they accrete. In all other areas, the retreat is greater for higher SLRs (Figs. 6, 7B–D,F).

In some areas of CLNS, shoreline retreat distances for the higher SLRs approach and often exceed the current island width (e.g., Fig. 7B–F), implying long-term barrier island rollover and recession landward, or the possibility of island breaching in areas with highly variable erosion rates. As sea level rises, the projected new shoreline locations show us the oceanward edge of the islands’ new locations. This landward barrier island migration indicates the flooding and groundwater results, especially for the higher SLRs with more migration, will occur in different locations than shown.

### Vertical land motion

**Fig. 8:**
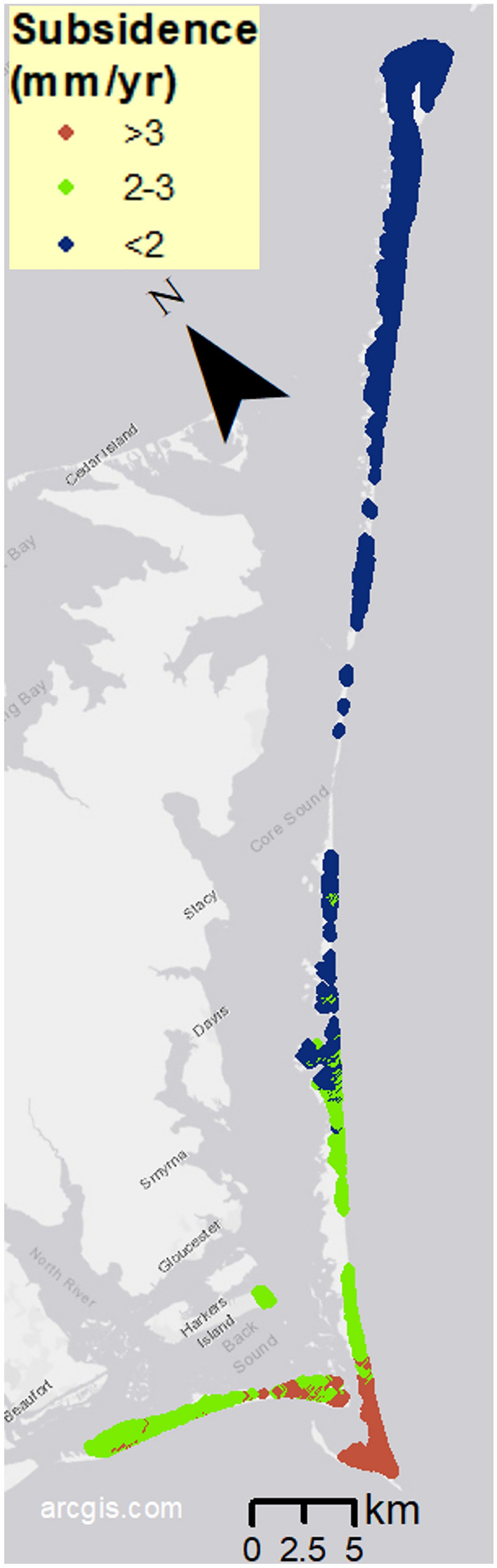
Map of satellite derived binned vertical land motion rate in mm/yr, indicating subsidence along all of Cape Lookout National Seashore. We created the map using ESRI ArcMap 10.8.1 software http://desktop.arcgis.com/en/arcmap.

Satellite-derived vertical land motion (VLM) for the years 2007 to 2020 at CLNS is universally negative, with subsidence rates increasing in magnitude to the south (Fig. 8). Subsidence rates have been binned into < 2 mm/yr; 2 to 3 mm/yr; and > 3 mm/yr, the most extreme subsidence for the region. Across CLNS, 34% of the subaerial land is subsiding by more than 2 mm/yr, including the eastern end of Harkers Island, where the Park Headquarters is located. The southernmost part of the region is subsiding by as much as 4.0 mm/yr. The other 66% of the region is subsiding at rates between 1.5 and 2.0 mm/yr. This sinking of the land increases relative SLR, which can affect estimated timing of SLR scenarios and their impacts.

## Discussion

For the present-day subaerial islands, focusing on the 0.5 m and 1.5 m SLR scenarios, those closest to the local estimated SLRs for the intermediate-high scenario for the years 2050 (0.46 m) and 2100 (1.60 m)^14^, respectively, the flooding model projects that close to half of the current land of CLNS will be inundated by 2050 and 84% by 2100. By 2050, annual storms could flood an additional 27% of land, for a total of 74% of the current land covered in water; and by 2100, 92% would be covered in water during an annual storm. Sound-side flooding of CLNS (Fig. 3) is a significant hazard, and it could drive further geomorphic changes not addressed in these studies^34^. The main concern with groundwater depths here is the projected loss of groundwater with SLR, including the loss of deep (> 2 m) groundwater areas, which are projected to decline from ~ 6 km^2^ presently to ~ 3 km^2^ in 2050 and ~ 1 km^2^ in 2100. Flooding from emergent groundwater here is not projected to be an extensive hazard in itself because marine/tidal inundation is projected to overtake most areas on CLNS where shallow groundwater would emerge. Average model estimates for shoreline retreat for the years 2050 and 2100 suggest that in narrower island regions, the retreat could be around 20–30% and 50–80% of the island width, respectively. The land subsidence of CLNS could accelerate the timing of each of these hazards. For example, a subsidence rate of 4 mm/yr in 100 years would raise relative sea level by 0.4 m, and therefore hazard exposure projections expected by 2100 could occur several decades earlier.

The projected shoreline positions and unmodeled changes in barrier island locations and morphology may increasingly change the patterns and total areas of flooding and groundwater depth categories with SLR. There have been successful attempts on modeling combined morphologic change and storm flooding on barrier islands^44^, but they were over short time scales. Modeling long-term morphologic change remains hindered by lack of long-term site-specific data and computational efficiency^17^.

The shoreline-change projections presented here do not explicitly parameterize processes related to long-term barrier-island migration, e.g., overwash, breaching, and inlet sediment dynamics, but instead captures these processes implicitly via the assumption of equilibrium-profile migration. As modeling advances are made, including changes in barrier island position and morphology in these projections will greatly improve estimates. A few very recent studies^21,22^ are getting closer to bridging the gap between idealized/exploratory and spatially-explicit/predictive models for barrier system dynamics. Future versions of CoSMoS-COAST are working toward explicitly including coupled beach-dune dynamics.

Modeling advances could also improve the groundwater modeling. The shallow groundwater levels calculated here used a simple representation of the geology and hydrology (see^43^ for details on model limitations), so a more detailed study incorporating additional hydrologic complexity, including salinity, could provide further understanding of fresh groundwater availability and the response of the water table. For example, increases in annual precipitation, from more severe storms, and from hurricanes^45^ are likely to lead to more groundwater recharge that is not included in the current models, and additional complexities associated with evapotranspiration, geologic heterogeneity, and salinization processes could diminish freshwater resources.

CLNS, while a largely undeveloped, natural system of barrier islands, still has many ecological, cultural, and historical resources to manage as the islands evolve. As the natural processes of barrier island migration are allowed to occur on CLNS, this will act to help preserve the barrier islands as well as some of the numerous ecological features there, including nesting areas and habitat for federally protected and/or threatened species such as piping plovers, American oystercatchers, and sea turtles, as well as the wild horses on Shackleford Banks. Preserving the barrier islands also means they will continue to be the first line of defense to the mainland coast against flooding and erosion. Although the barrier islands can naturally migrate landward due to SLR, the cultural and historic resources they contain cannot. There are culturally valuable features such as the historical Portsmouth Village at the north end of North Core Banks and the historical Cape Lookout Village. The Cape Lookout Lighthouse, while also a historic feature, is still an operational aid to navigation. There are two areas for cabin camping on the islands—Great Island cabins on South Core Banks and Long Point cabins on North Core Banks—and primitive tent camping is available along the beaches of the islands. There are several ferry landings to access these features.

Even at present-day sea level, storms are already severely impacting the islands of CLNS and its many resources^34^ (Fig. 2, dark blue boxes; Fig. 3A). In fact, the Long Point Cabins of CLNS were so severely damaged from Hurricane Dorian (2019) that the NPS has determined they cannot sustain the cabins at their current location and thus they plan to demolish the damaged structures^34,42,46,47^. The model results estimate that by 2100, the shoreline near the Long Point cabins may have retreated landward by over half the island width, to around the present-day ferry landing location on the sound side of the island (Fig. 7B). This would suggest significant morphological change and migration of the island landward, with the island potentially no longer present in the area of the damaged Long Point cabin infrastructure. Shackleford Banks appears to remain the most stable of the barrier islands across flooding, groundwater, and shoreline change hazards (Figs. 3, 5, 7F), however the increased rate of subsidence here could increase hazard exposure. The horses on Shackleford Banks rely on access to fresh drinking water from freshwater ponds and groundwater that they reach by digging^46^, so the aforementioned future model improvements would help provide further understanding of the fresh groundwater availability to the horses.

A challenge for the National Park Service is balancing its mission to protect cultural and historic structures and sites while allowing natural processes such as barrier island retreat and overwash to occur. For example, piping plovers have shown a preference for overwash habitats^48^, but overwash can threaten cultural and historic structures. Managing a diverse array of resources is a common challenge for the agencies tasked with managing barrier islands across the world, and just as on CLNS, requires a wide range of expertise, tools, and decisions to be made to balance the various hazard impacts and budgetary constraints of the agency. The challenge to implement sustainable coastal management practices in a dynamic coastal environment will become exacerbated due to accelerating SLR and storm impacts, and decisions about repairing damage to ecosystems and infrastructure would benefit by considering the combined increased future hazard risks from SLR and storms. Coastal land managers globally will increasingly grapple with the difficult decision to protect vs. relocate coastal resources in the coming decades.

## Materials and methods

### Overland flooding

Overland flood hazards were simulated with the open-source Super-Fast INundation of CoastS (SFINCS) model^49^. SFINCS is a computationally efficient reduced complexity model that approximates the shallow water equations, but at a lower computational expense than a traditional full-physics model^50^. For CLNS, the study area was modeled using one ~ 300 km × 200 km model domain from Norfolk to Wilmington in the north–south direction and from the approximate + 10 m NAVD88 landward elevation contour to the -5 m NAVD88 isobath. Remaining sections of the South Atlantic Coast were simulated with four additional overlapping model domains that were merged to create continuous outputs^38^. The model was run with a grid resolution of 200 m by 200 m in combination with subgrid lookup tables developed from a 1 m resolution topo-bathymetric dataset (Coastal National Elevation Database, CoNED)^51,52^. Dune heights in the baseline model grid were set to maximum elevations found in the CoNED data within a grid cell containing dunes and cross-checked with Doran et al.^53^.

Spatiotemporally varying time-series of water levels, wave setup, fluvial discharges, and rainfall were applied at the boundaries of the SFINCS model for tens of thousands of emulated extratropical and tropical storms. In contrast to previous CoSMoS work^27^, no design event based on offshore conditions is used but flooding for extratropical and tropical simulations were combined on grid cell basis and thus provide a more accurate description of the flood hazards, as hazard and return period are assessed locally at each grid cell. Open boundary water levels (astronomic tides, storm surge, and wave setup) associated with tropical cyclones (TCs) (hurricanes) were derived from the U.S. Army Corps of Engineers (USACE) Coastal Hazards System (CHS)^54–57^ database of TCs. For extratropical events, the Global Tide and Surge Model (GTSM)^58–60^ outputs and externally computed wave setup were used^39^.

The CHS is a hybrid probabilistic-dynamic model using the joint probability method and statistically/dynamically downscaled storm surge, tides, waves, and wave setup. Wave setup for all non-hurricane events were computed with the empirical Stockdon^61^ formulation using local foreshore beach slopes^62^ and WaveWatch3 (WW3) simulated waves^63^. The GTSM and WW3 models were forced by three global climate models (GCMs) from the High-Resolution Model Intercomparison Project (HighResMIP, 6th generation Coupled Model Intercomparison Project – CMIP6) that represent the ‘high’ emissions scenario SSP5-8.5 for the projection period (2020–2050). The HighResMiP models CMCC-CM2-VHR4^64^, GFDL-CMC4C192^65^, and HadGEM3^66^ were chosen because of the finer atmosphere and ocean grid resolutions (25–50 km) which better resolve coastal storm events compared to previous GCMs (O(100kms))^67,68^ and have a similar resolution to the European Centre for Medium-Range Weather Forecasts Reanalysis-5 (ERA5)^69^. Rainfall was obtained directly from GCM outputs and for the TC runs were based on the Interagency Performance Evaluation Task Force Rainfall Analysis^70^ method.

Further information on model setup and validation is detailed in Nederhoff^38^. The physics-based hydrodynamic modeling used for the flood assessment can skillfully reproduce coastal water levels based on a comprehensive validation of tides, almost two hundred historical storms, and an in-depth hindcast of Hurricane Florence^38^. The validations nearest our study region have a mean-absolute-error (MAE) of 8.3 cm for tide and 14.7 cm for storms at the Oregon Inlet Marina, NC, and an MAE of 6.5 cm for tide and 9.7 cm for storms at Beaufort, NC^38^.

### Groundwater depths

Groundwater levels were simulated using steady-state MODFLOW-NWT^71^ groundwater flow models after the methodology used in CoSMoS applied to California^43^. The response of the water table depth to SLR was modeled without identifying the salinity of the groundwater. For the CLNS, only one groundwater model domain spanned the full study area, whereas other regions of the full U.S. Southeast model domain included overlapping models that were merged to create continuous outputs. The groundwater model was developed using a 50 m by 50 m square grid cell size in the horizontal and a variable thickness set by a previous groundwater flow model in the area^72^, based on calibrated geologic structure which used an inverse process to adjust the aquifer thickness and hydraulic conductivity of the shallow aquifer to match observed well levels as closely as possible. The hydraulic conductivity in each grid cell was assigned by dividing the calibrated transmissivity by the saturated thickness modeled by Zell and Sanford^72^. The top model surface, the ground surface, was set to the topography, down-sampled from the 1-m elevation model used for the overland flood hazards^51,52^. This surface of the model had of two boundary conditions: 1) recharge prescribed by average annual recharge derived from 2000–2013 observations and empirical regression equations^73,74^ (RCH MODFLOW package), and 2) a drain condition with an elevation set to the topographic elevation of the grid cell and a conductance set by the grid cell size and the cell hydraulic conductivity (DRN MODFLOW package).

Full CoSMoS water level outputs were not available at the time of the groundwater modeling, so the marine boundary condition, set as a constant head cell (CHD MODFLOW package), was assigned the nearest elevation from the gridded NOAA VDATUM Mean Higher High Water dataset^75^ for present day and raised by a constant amount of SLR for higher sea level scenarios in separate model instances. Future research should directly include dynamic marine boundary conditions from CoSMoS flood modeling into the groundwater modeling, which would help determine the full transient nature of the groundwater hazard, such as during storms. This improvement would come at a significant cost in increased modeling complexity and has not yet been implemented. Water table depth was calculated at a 10 m by 10 m resolution using the modeled hydraulic heads subtracted from the DEM.

### Shoreline Change

Shoreline change was modeled with CoSMoS-COAST^76–78^, which solves the one-dimensional conservation of sediment volume in the alongshore direction that is given by:

1 $$\underbrace {{\frac{\partial Y}{{\partial t}}}}_{shoreline change} = \overbrace {{\underbrace {{ - \frac{1}{{d_{c} }}\frac{\partial Q}{{\partial X}}}}_{{\left\{ 1 \right\} longshore transport}} - \underbrace {{\frac{c}{\tan \beta }\frac{\partial S}{{\partial t}}}}_{{\begin{array}{*{20}c} {\left\{ 2 \right\} shoreline migration} \\ {due to sea - level rise} \\ \end{array} }} + \underbrace {{\upsilon_{lt} }}_{{\begin{array}{*{20}c} {\left\{ 3 \right\} long - term residual } \\ {shoreline trend;} \\ {unresolved processes} \\ \end{array} }}}}^{long - term processes} + \overbrace {{\underbrace {{\frac{1}{\tau }\left( {Y_{eq} - Y} \right)}}_{{\begin{array}{*{20}c} {\left\{ 4 \right\} cross - shore } \\ {^{\prime}equlibirum^{\prime} } \\ {transport} \\ \end{array} }} + \underbrace {\varepsilon }_{{\left\{ 5 \right\} additive noise}}}}^{short - term processes}$$

As discussed in Vitousek et al.^78^, Eq. (1) synthesizes several popular individual-process models including: {1} a ‘one-line’ model for longshore transport; {2} a cross-shore beach profile change model due to sea-level rise; {3} a long-term residual shoreline trend that represents sources and sinks of sediment, {4} a wave-driven cross-shore equilibrium shoreline change model, and finally {5} a noise term.

The South Atlantic model is comprised of roughly 34,000 transects (spaced every 50 m in the alongshore direction), which span open-coast sandy beaches on the U.S. South Atlantic Coast from Miami, Florida, to Cape Henlopen, Delaware. The portion of the model covering CLNS is comprised of 1,593 transects. The model was forced with daily hindcasted and projected wave conditions obtained from the ERA5 reanalysis and 7 future wave scenarios from the CMIP6 collection of GCMs, respectively, thus including stochasticity of storm dynamics. The model was calibrated via an ensemble Kalman filter using a 200-member model ensemble and 25 years of satellite-derived shorelines observations (from 1990–2015 and spanning the entire model domain) obtained using the CoastSat toolbox^79^, thus implicitly accounting for the effects of local dune dynamics on shoreline position. After the calibration period, the model was validated using 5 years of satellite-derived shoreline observations (from 2015–2020). The validation period assesses that the model achieves a median root-mean-square accuracy of 11.5 m across CLNS and 12.3 m across the entire South Atlantic domain, respectively.

The model currently only keeps track of one coastal feature (i.e., the Mean Sea Level shoreline contour; variable *Y* in Eq. (1)), and assumes that other morphologic features, e.g., dune toe and crest positions and back barrier shoreline, remain in equilibrium with this reference feature. Other long term coastal morphology models account for the coevolution of many components of barrier island systems (e.g., shoreface, barrier/dune, marsh, and lagoon; see review by Hoagland et al.^17^), with a small subset of models including all aforementioned barrier-island components^32,80,81^. But, these long-term barrier-island models, which were originally created to explore morphodynamics, have largely remained exploratory^17^. The limited availability of spatially and temporally dense observations of coastal features such as dune toe, dune crest, and back barrier shoreline, which are needed to understand and model how these features change over time, is a limiting factor for “predictive” rather than “exploratory” modeling of barrier islands, based on the definitions in Murray^82^. As datasets of coastal profile elevations, such as Doran et al.^53^, become more spatiotemporally dense, models can begin to assimilate and integrate these features into predictions.

Recent remote sensing methods^79^ have provided tremendous resources for historical shoreline positions, which represent the basis for the data-driven future projection modeling of the current application. Many modern barrier island migration models rely on equilibrium profile transgression^17,83^, which implicitly accounts for processes such as overwash. A limitation to this model, and to most long-term barrier island transgression models, is that it does not consider the effect of breaches, which are possible due to SLR in the future. If temporary breaches fill in over time^34^, then the model results presented here should be a robust estimate despite this unavoidable uncertainty. More information on the model and validation is detailed in^40^.

### Vertical land motion

We employed an advanced multitemporal synthetic aperture radar (SAR) processing algorithm to determine VLM using data obtained from the ascending orbit of Sentinel-1 A/B and ALOS-1 satellites. Initial steps involved the creation of line-of-sight (LOS) velocity maps for Sentinel-1 A/B and ALOS-1 satellites using a multitemporal wavelet-based InSAR (WabInSAR) algorithm^84–87^. We used GAMMA software^88^ to generate interferograms. Interferograms were corrected for the effect of topography using the Shuttle Radar Topography Mission (SRTM)^89^ 30 m digital elevation model (DEM). Elite (i.e., less noisy) pixels were obtained by applying a threshold of 0.7 on the temporal averaged coherence of each pixel. To estimate the absolute phase change of elite pixels, we applied a minimum cost flow algorithm^90^, suitable for sparsely distributed pixels^91^. Next, we corrected unwrapped interferograms for the effect of orbital error^92^, topographically correlated atmospheric phase delay, and spatially uncorrelated DEM error^77,78^. We applied a reweighted least-squares optimization to resolve each pixel’s Line-of-Sight (LOS) time series and velocity^85^. To generate the 3D velocities tied to the International Global Navigation Satellite Systems (GNSS) Service reference global frame of 2014 IGS14, we used a stochastic model^93^ to combine the LOS velocities with the observation of GNSS. The validation test against independent GNSS measurements yields an accuracy better than 1 mm/yr for the VLM rate within the IGS14 reference frame^94^. Ohenhen^94^ has published VLM data from 2007 to 2020, encompassing the study area with continuous spatial coverage at a 50-m resolution.

## Data Availability

All data used in this publication are available from: Barnard, P.L., Befus, K.M., Danielson, J.J., Engelstad, A.C., Erikson, L.H., Foxgrover, A.C., Hardy, M.W., Hoover, D.J., Leijnse, T., Massey, C., McCall, R., Nadal-Caraballo, N., Nederhoff, K.M., Ohenhen, L., O'Neill, A., Parker, K.A., Shirzaei, M., Su, X., Thomas, J.A., van Ormondt, M., Vitousek, S.F., Vox, K., and Yawn, M.C., 2023, Future coastal hazards along the U.S. North and South Carolina coasts: U.S. Geological Survey data release, https://doi.org/10.5066/P9W91314. Each of the datasets shown in this publication were downloaded from the above source, and then reduced to just present the CLNS region (Fig. 1) for the following specific products: projections of coastal flood hazards and flood potential; projected groundwater emergence and shoaling; projections of shoreline change of current and future (2005–2100) sea-level rise scenarios (case 4, shoreline positions are allowed to move landward without limitation, and the model assumes recent historical accretion rates continue into the future; slope B, intermediate slope); and vertical land motion rates for the years 2007 to 2020. The boundary for Cape Lookout National Seashore came from the National Park Service nps boundary ArcGIS open dataset at https://public-nps.opendata.arcgis.com/datasets/nps::nps-boundary-1/explore.
